# HIV Detection and Delayed Diagnosis: A Time Series Analysis in China

**DOI:** 10.3390/ijerph192416917

**Published:** 2022-12-16

**Authors:** Junfang Chen, Junfang Xu, Yuyin Zhou, Yan Luo

**Affiliations:** 1 Hangzhou Center for Disease Control and Prevention, Hangzhou 310021, China; 2 Center for Health Policy Studies, School of Public Health, Zhejiang University School of Medicine, Hangzhou 310058, China; 3 Department of Pharmacy, Second Affiliated Hospital, Zhejiang University School of Medicine, Hangzhou 310009, China; 4 Shenzhen Pingshan District Center for Disease Control and Prevention, Shenzhen 518118, China

**Keywords:** HIV detection, delayed diagnosis, China

## Abstract

Background: Insufficient HIV detection and late presentation to antiretroviral therapy (ART) pose significant public health challenges. In China, universal access to HIV testing is available now. Under this background, we aim to analyze the trends of HIV detection and the prevalence of delayed HIV diagnosis (DHD) in order to provide evidence for HIV prevention and treatment in China. Methods: Data of HIV tests in Hangzhou city between 2007 and 2018 were collected from the Chinese National HIV/AIDS Comprehensive Response Information Management System (CRIMS). Descriptive statistics were used to describe the characteristics of HIV testing and detection and the prevalence of DHD among newly diagnosed HIV cases. Non-parametric tests were employed to examine the prevalence of DHD among newly diagnosed HIV cases. Moreover, logistic regression models were employed to explore the influencing factors of DHD. Results: Testing rates doubled from 14.1% in 2007–2010 to 28.2% in 2016–2018. The total positive rate of HIV tests was 5.3 per 10,000. Preoperative testing was the predominant pathway for HIV tests, accounting for 41.9%, followed by testing for health screening, maternal examination and other patients, accounting for 18.4%, 13.2% and 11.8%, respectively. Meanwhile, the predominant pathway for HIV case detection was also preoperative testing, accounting for 29.1%, followed by testing for other patients, testing at STD clinics and VCT, with the proportions of 18.8%, 15.8% and 13.6%, respectively. MSM (men who have sex with men) contact was the main transmission route, accounting for 55.3%, followed by heterosexual contact, accounting for 41.6%. Overall, DHD occurred in 29.0% of the newly diagnosed cases, and this rate had not improved over the years. A higher prevalence of DHD was found in those diagnosed through a pre-test for receiving blood/products [OR (95%CI): 5.42(2.95–9.97)], detection of other patients [OR (95%CI): 2.08(1.64–2.63)], preoperative testing [OR (95%CI): 1.83(1.44–2.32)] and spouse or sexual partner testing in positive person [OR (95%CI): 1.93(1.34–2.78)] compared with those diagnosed at a VCT clinic. Heterosexuals [OR (95%CI): 1.20(1.06–1.36)] had a higher risk of DHD than MSM. Diagnosis at a CDC [OR (95%CI): 0.68(0.55–0.83)] and community health centers [OR (95%CI): 0.54(0.39–0.75)] had a lower risk of DHD than in hospitals. Older age, males, being single/divorced or widowed and floating population were also associated with DHD. Conclusions: In China, DHD had not improved in the last 10 years, although HIV testing had been expanded. Therefore, it is important for continued efforts to promote early diagnosis of HIV to prevent transmission, morbidity and early mortality in HIV infection.

## 1. Introduction

Worldwide in 2019, 38 million people were reported as living with HIV/AIDS and 1.7 million were newly diagnosed [1,2]. In China, the number of people living with HIV/AIDS was up to 958,000 in the year 2019 [1,3]. However, it is estimated these HIV-positive people only accounted for 68.9% of the total people living with HIV/AIDS in China [1]. Nonetheless, more than 30% are undiagnosed and did not know their HIV status, which is almost twice the corresponding proportion of 15% in the United States [1,2]. However, awareness of HIV infection status is crucial to the reduction of transmission and the incidence of HIV epidemic. Previous studies have shown that more than 50% of newly diagnosed infections can be attributed to transmission from those with undiagnosed HIV infection [4]. It is reported that the rate of advanced HIV infections among newly diagnosed cases in China ranged from 35.5% to 42.1% [5]. For individuals, delayed HIV diagnosis (DHD) caused people to not access life-saving anti-retroviral therapy early in order to prevent HIV-associated morbidity and mortality [6]. From a population perspective, DHD represents missed opportunities to prevent increasing HIV transmission [7]. Therefore, DHD is detrimental both to the individual and to society [6]. Targeted strategies to promote case detection and early diagnosis of HIV infection are crucial. HIV testing is considered key to early HIV care, treatment and prevention. As a result, China’s 13th Five-Year Plan of Action to Curb and Prevent AIDS incorporated the objective to increase the percentage of people who know their HIV status to 90% [8]. Under this background, we aim to analyze whether DHD improved in the past 10 years with universal HIV testing.

## 2. Materials and Methods

### 2.1. Data Source

All the data, including HIV tests, diagnosis and positive cases, were collected from the Chinese National HIV/AIDS Comprehensive Response Information Management System (CRIMS) of Hangzhou City between 2007 and 2018. Hangzhou is the capital city of Zhejiang Province, located in the southeast of China, and it is also the first city to report local Chinese HIV/AIDS cases infected by using import factor Ⅷ in 1985. The epidemic of HIV/AIDS in Hangzhou experienced dissemination, diffusion and a rapid growth stage since 1985, which was consistent with the whole of China. In order to promote early diagnosis and treatment, Hangzhou issued a policy of free HIV testing for preoperative patients and pregnant women in 2007 following the national policy call. The information on HIV newly diagnosed cases included age, date of HIV diagnosis, gender, marital status, occupation, education level, ethnicity, permanent residence, route of HIV transmission, testing route and venues, CD4 T cell count at diagnosis and year of diagnosis.

### 2.2. Variable Definition

Testing routes were classified into 10 types, including preoperative testing, pre-test of receiving blood/products, testing at sexual transmitted diseases (STD) clinics, testing of other patients, maternal examination, voluntary counselling and testing (VCT), spouse or sexual partner testing in positive person, health screening, physical examination of detainee and others. Testing venues included hospital settings, disease control and prevention institutions (CDC), community health centers (CHCs) and blood centers. Delayed HIV diagnosis (DHD) was defined as a person diagnosed with HIV with a CD4 cell count below 200 cells/ul or presenting with an AIDS-defining illness, regardless of the CD4 cell count, in the six months after diagnosis [9].

Before 2010, all of the HIV-antibody positive samples should have been sent to the HIV antibody confirmation laboratory for further confirmatory tests in Hangzhou. Therefore, we calculated the positive rate of the tests directly using the number of confirmed positives as numerator and the total number of tests as denominator. Since 2010, HIV-antibody positive samples for the primary screening would be duplicate checked before further confirmatory tests, and if HIV was diagnosed, confirmatory tests would not be conducted again. Consequently, diagnosed cases were only recorded in the numbers of primary screening positive, and they were not recorded in the numbers of confirmed positive in our collected data. As a result, we calculated the numbers of confirmed positive by the numbers of screening positive, multiplying the proportions of primary screening positive and the proportions of confirmatory positive in the same year. The proportions were obtained from the HIV-antibody Confirmatory Laboratory in Hangzhou CDC, and they were calculated respectively for blood centers and other kinds of testing institutions to adjust the numbers of confirmed positive reported by the corresponding kind of testing institution.

### 2.3. Statistical Analysis

Descriptive statistics was used to describe the trends of HIV testing, the prevalence of DHD and the characteristics of newly diagnosed HIV cases for the overall population and different subgroups in the last 10 years. Trends of positive HIV cases by transmission routes and baseline CD4 cell count from 2007 to 2018 were also described. Non-parametric tests were employed to examine the differences of HIV testing in the last 10 years and the baseline CD4 cell count by different characteristics (e.g., age, gender, marital status, education, ethnicity, residence, testing route, venues and transmission routes) of newly diagnosed HIV people. Logistic regression models were employed to explore the influencing factors of DHD, and the independent variables included year, age group, gender, marital status, education, occupation, ethnicity, regional categories, permanent residence, detection route, detection venues and transmission route. The statistical software SPSS 23.0 (IBM, Armonk, NY, USA) was used to analyze all the data. Values with a two-tailed significance level less than 0.05 were considered statistically significant.

## 3. Results

### 3.1. Characteristics of HIV Tests and Diagnosis

As seen in Table 1, both the number of HIV screening tests and diagnosed HIV cases showed an obvious increasing trend from 2007 to 2018. Testing rates (=the number of people receiving test/the total number permanent population) doubled from 14.1% during 2007–2010 to 28.2% during 2016–2018. The positive rate of HIV tests was 5.3 per 10,000 in total, and it increased rapidly from 2.8 to 7.4 per 10,000 in the last 10 years. HIV screening tests and cases diagnosed mainly concentrated in the downtown district, accounting for 83.6% and 92.7%, respectively, and the positive rate in the downtown district was much higher than in the suburbs (5.9 vs. 2.4 per 10,000). Preoperative testing was the predominant pathway for HIV screening tests, accounting for 41.9%, followed by testing for health screening, maternal examination and other patients, accounting for 18.4%, 13.2% and 11.8%, respectively. Meanwhile, the predominant pathway for HIV case detection was also preoperative testing, accounting for 29.1%, followed by testing for other patients, testing at STD clinics and VCT, with the proportions of 18.8%, 15.8% and 13.6%, respectively. The trends of the absolute testing number of the main routes of HIV screening tests or case detection showed that the volumes of preoperative testing, testing of other patients and maternal examination increased greatly during the period, and the volumes of testing at STD clinics experienced a smaller increase.

The HIV-positive rate for spouses or sexual partners of positive people reached 818.3, which included 63.9 for testing at VCT clinics, 21.5 for testing at STD clinics and 12.9 for physical examination of detainee per 10,000, respectively. From the perspective of different testing institutions, HIV screening tests predominantly offered by hospital settings accounted for 73.6%, with a proportion of 66.0% for case detection. Then, 14.4% and 9.4% of tests offered by CDCs and blood centers, respectively, followed, with a proportion of 29.2% and 3.6% for cases diagnosed, respectively. The positive rate of testing at CDCs (10.8 per 10,000) was much higher than others, including hospital settings, CHCs and blood centers (4.8, 2.5 and 2.0 per 10,000, respectively).

### 3.2. Transmission Routes among HIV Newly Diagnosed Cases

**Figure 1:**
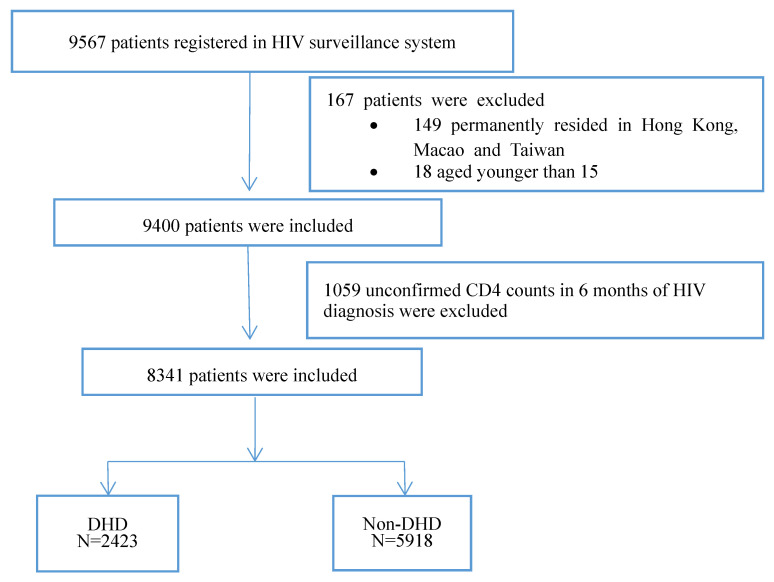
Flow chart of DHD analysis.

**Figure 2:**
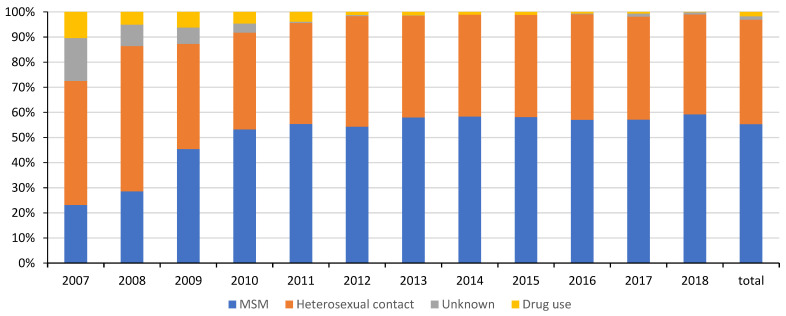
Trends of distribution of transmission routes of HIV newly diagnosed cases in Hangzhou, 2007–2018.

A total of 9567 patients with newly diagnosed HIV registered in the HIV surveillance system of the Hangzhou CDC between 2007 and 2018. Among them, 9400 patients permanently resided in mainland China and were age 15 years or older (Figure 1). As shown in Figure 2, the numbers of newly diagnosed HIV cases increased rapidly from 164 in 2007 to 1204 in 2015. The number has remained stable at approximately 1200 cases per year since then, with a total of 9400 during 2007–2018, while the cases of both MSM (men who have sex with men) and heterosexual contact increased rapidly each year. MSM contact was the main transmission route of HIV infection in Hangzhou, accounting for 55.3%, followed by heterosexual contact with 41.6%. The percentage of MSM transmission soared from 23.17% in 2007 to 45.40% in 2009, and then continuously increased to 59.23% in 2018. On the contrary, the percentage of heterosexual contact increased from 49.39% in 2007 to 57.86% in 2008, and dropped rapidly to 41.89% in 2009, and it has remained around 40% since then.

### 3.3. Baseline CD4 Count and DHD

As shown in Table 2, the overall proportion of HIV cases receiving treatment with a baseline CD4 cell count within 6 months of diagnosis was 88.7%, representing an obvious increase from 67.4% during 2007–2009 to 93.8% during 2016–2018. In total, nearly 60% of the patients (N = 4822, 57.8%) had baseline CD4 cell counts < 350 cells/uL, 2389 patients (28.6%) had baseline CD4 cell counts < 200 cells/uL and less than 20% of patients (N = 1461, 17.5%) had baseline CD4 cell counts ≧500 cells/uL.

**Figure 3:**
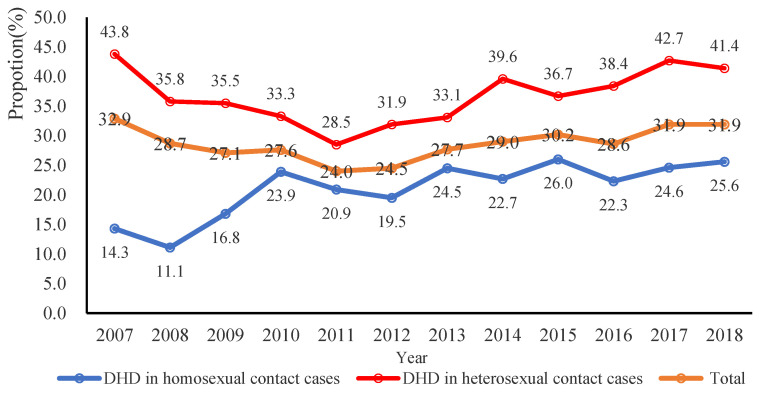
Trends of DHD among HIV newly diagnosed cases in Hangzhou, 2007–2018.

As shown in Figure 3 and Table 3, the proportion of subjects with DHD was 29.0% in total, and it decreased from 32.9% in 2007 to 24.0% in 2011 and then increased to 31.9% in 2018. DHD in heterosexual contact cases was much higher than in cases with MSM contact or injecting drug use, accounting for 37.4%, 23.3% and 22.4%, respectively. In general, the proportion of DHD in the MSM transmission cases showed an increasing trend, from 14.3% in 2007 to 25.6% in 2018, while that in the heterosexual contact cases decreased from 43.8% in 2007 to 28.5% in 2011, and thereafter increased to 41.4% in 2018.

With the increase of age, the proportion of HIV people with DHD increased from 10.4% in the age group below 20 to 45.4% in the age group 50 and above. The proportion of DHD in the single/divorced/widowed HIV cases was lower than that in the married cases (24.2% vs. 40.8%). With the increase of educational level, the proportion of DHD declined, from 40.1% for primary school to 22.4% for college or above. Retired personnel and farmers had a relatively higher proportion of DHD (45.3% and 43.6%, respectively). The proportion of DHD in migrants was higher than that of local residents (32.8% vs. 26.8%). HIV screening tests integrated in the physical examination of detainees, pregnant women and healthy people led to the lowest proportions of DHD, and cases diagnosed at VCT also had a relatively lower proportion of DHD (16.8%). The proportion was of average level among those diagnosed via STD clinics (27.0%), but substantially higher for pre-test to receive blood/products (69.5%), preoperative testing (41.8%), testing of other patients (41.5%) and spouse or sexual partner testing in positive person (38.7%). In terms of detection venues, the proportion of DHD among cases diagnosed at hospital settings (36.7%) was substantially higher than other institutions, including CDCs (18.1%), community health centers (15.8%) and blood centers (13.2%).

### 3.4. Factors Associated with DHD

In Table 3, the multivariate logistic regression model showed that the increase of age was statistically associated with DHD, with AOR of 1.38 (95%CI 1.31–1.46) compared to the reference category of <20 years. Males had a higher odds of DHD (AOR 1.53; 95%CI 1.29–1.82) than females. Married people had a higher odds of DHD (AOR 1.23; 95%CI 1.08–1.39) compared with single/divorced/widowed people. The migrant population had a higher odds of DHD (AOR 1.23; 95%CI 1.11–1.37) than local residents. Compared with cases diagnosed through VCT, those diagnosed through a pre-test for receiving blood/products (AOR 5.42; 95%CI 2.95–9.97), testing of other patients (AOR 2.08; 95%CI 1.64–2.63), preoperative testing (AOR 1.83; 95%CI 1.44–2.32) and spouse or sexual partner testing in positive person (AOR 1.93; 95%CI 1.34–2.78) also had a higher odds of DHD, while cases diagnosed through the physical examination of detainees were the opposite (AOR 0.64; 95%CI 0.42–0.99). Being diagnosed at CDCs (AOR 0.68; 95%CI 0.55–0.83) and community health centers (AOR 0.54; 95%CI 0.39–0.75) had a lower odds of DHD. Cases with a heterosexual contact history also had a higher odds of DHD (AOR 1.20; 95%CI 1.06–1.36) than those with a MSM contact history.

## 4. Discussion

The HIV testing rate in Hangzhou doubled from 14.1% during 2007–2009 to 28.2% during 2016–2018. After the implementation of AIDS prevention and control regulations in Zhejiang Province in 2007, hospitals in Hangzhou began to provide free testing for every patient intended to have a surgical intervention or invasive diagnostic procedure, as well as every pregnant woman, and testing volumes for the populations increased obviously over the years. In 2013, Hangzhou participated in the national pilot project for expanding testing. We enhanced HIV testing led by clinicians for STD clinic attendants and inpatients, and the screening requirements for STD clinic attendants were included in the assessment requirements of health administrative departments for local medical institutions. Since the start of the pilot project, screening for STD clinic attendants and other clinical patients increased effectively, playing an important role in HIV case detection. In general, HIV testing expansion in Hangzhou during 2007–2018 was mainly due to the increase of provider-initiated HIV testing at hospital settings, especially for preoperative testing, other patients including inpatient, clinical suspects testing, maternal testing and testing at STD clinics, most of which was mandatory. Several studies have shown that offering routine screening is associated with an increased uptake of testing, especially when testing is recommended by a healthcare provider. The volumes of both HIV screening tests and HIV cases detection via hospital settings made up the vast majority, and the obvious HIV testing increments of patients presented in clinical settings indicated that awareness of HIV testing among healthcare providers is improving. Moreover, provider-initiated HIV testing expanded and played an important role in HIV testing and case detection in Hangzhou city.

The mode of HIV transmission in Hangzhou experienced great changes during the period. The proportion of newly diagnosed cases with MSM transmission exceeded that with heterosexual transmission in 2009, and it became the predominant transmission route. This is consistent with previous studies indicating that sexual transmission has become the dominant mode of HIV transmission in Zhejiang province and even in China [10,11]. What is different, however, is that MSM transmission is the leading route of HIV infection in recent years in Hangzhou, while heterosexual transmission was still the leading route at the national level. Men who have sex with men (MSM) and IDU had the lower proportions of DHD. This suggests that outreach efforts to reach these high-risk populations produced some success in promoting early and regular HIV screening [11].

Moreover, our results were similar to previous studies that indicated the proportion of people with late diagnoses is stable or worsening internationally [12]. Overall, 29.0% of people were diagnosed with DHD. The overall proportion of DHD was lower than the national level and higher than the provincial level [12]. DHD showed a decreasing trend until 2012, and then increased thereafter. Therefore, despite the dramatic expansion of HIV testing, it did not reduce the proportion of DHD. In addition, the expansion of testing predominantly constituted clinic testing, which included more illness-triggered testing and led to a relatively higher proportion of late diagnoses. Testing integrated in various physical examinations and VCT, which can lead to earlier diagnosis, accounted for just a small proportion of the total testing volume, and the increment of such testing in the first six years was more obvious during 2007–2012 than that during 2013–2018. As a result, DHD decreased in previous years and then increased in the later years, and it was not improved, and even worsened a little, during the whole period despite the expansion of testing in general. Our findings suggest it is necessary to integrate HIV testing into physical examination for adults and further establish universal routine HIV testing as a standard of care for all people in private and public care settings, regardless of patient-reported HIV risk.

This study shows that cases diagnosed at CDCs and community health service centers were less likely to be diagnosed late compared with hospital settings. Meanwhile, cases diagnosed by clinic testing, such as pre-test of receiving blood/products, testing of other patients and preoperative testing, were more likely to be diagnosed late compared with those diagnosed by VCT. In Hangzhou, there were 24 VCT clinics affiliated with CDCs and CHCs, providing routine free HIV counseling and testing, while during the past six years, the volumes of VCT did not increase but decreased compared with previous years; thus, VCT should be expanded to facilitate early diagnosis. CHCs are important providers of primary and preventive care, and they are also the first gateway for patients to seek medical treatment. Rapid testing is available in all CHCs in Hangzhou, but the testing service is not fully implemented at the moment. Providing screening and care for HIV should be a priority in these primary settings because health center patients are disproportionately affected by HIV.

Cases with MSM transmission were at lower risk of DHD than those of heterosexual transmission, and this is in accordance with recent studies indicating that MSM present with higher median CD4 levels compared to other risk groups [13]. The relative success in earlier diagnosis in HIV-positive MSM may be due to the Gates Foundation Project and Global Fund AIDS Project since 2008. These projects cultivated local MSM community groups, and with the support of the community groups, comprehensive prevention, especially testing mobilization targeted towards MSM, was continuously reinforced. The projects led to policies and additional ongoing intervention programs aided by local government finance that address HIV prevention and HIV diagnosis among MSM. However, effective HIV testing strategies are lacking for heterosexuals. Consistent with the results of previous studies [14], our study revealed that men were associated with DHD, possibly because HIV-infected women generally experience a slower disease development compared with men, which was corroborated by the fact that women tended to have higher CD4 cell counts compared to men with a similar infection time [14]. We also found that older patients were more likely to present at late disease stages, and similar results have been widely reported [14,15]. An accepted explanation for the association with older age could be that older people, like other low-risk groups, have a lower perceived risk for HIV infection by both themselves and their health-care providers; therefore, they receive HIV testing later than younger people [16,17,18]. In addition, older people may perceive HIV symptoms as normal aging and not consider HIV as a cause [19]. Local residents were at lower risk for DHD, which might indicate the greater availability of HIV screening services provided to local residents compared to the floating population.

This study had some limitations. First, we calculated the confirmed positive numbers using screening positive numbers, which might produce bias. Nevertheless, the adjusted proportion was computed using the data of confirmatory tests of the whole Hangzhou district with enough sample size year by year, and we took the difference between blood centers and other testing institutions into account, all of which could partially reduce the bias. Second, we excluded 1059 people who had CD4 cell count data missing, which accounted for 11.3% of the total new diagnoses. Excluded people were more likely to have poor compliance and to die prior to having their CD4 cell count measured; thus, their exclusion likely resulted in an underestimation of late presentation. Third, the variables used in this study were somewhat limited because this study was a retrospective investigation; hence, some influencing factors might have been missed. Moreover, the overall probability of Type I errors in some results may be inflated, which needs a deep analysis.

## 5. Conclusions

HIV testing and case detection experienced a general expansion in Hangzhou during 2007–2018, but hospital-based testing plays a major role in HIV testing and case detection. Despite the overall expansion of testing, delayed diagnosis slightly increased. To expand HIV provider-initiated screening and to establish routine HIV testing as a procedure of healthcare in all medical settings are crucial to further expand HIV case detection and diminish delayed diagnosis.

## Figures and Tables

**Table 1:** HIV screening and diagnosis rate in Hangzhou, 2007–2018.

Items	2007~2009	2010~2012	2013~2015	2016~2018
Screening rate, % ^a^	14.1	19.8	21.6	28.2
Positive rate, %	2.8	2.7	6.4	7.4
Regional categories				
Downtown	3.0	2.9	7.2	8.1
Suburbs	1.2	1.5	2.4	3.4
Detection route				
Preoperative testing	2.0	1.6	4.2	5.2
Pre-test of receiving blood/products	0.6	0.4	2.4	3.1
Testing at STD Clinics	9.2	6.3	26.5	30.9
Testing of other patients ^b^	4.3	4.7	8.8	9.8
Maternal examination	0.5	0.5	1.2	0.9
VCT	27.8	28.0	90.3	115
Spouse or sexual partner testing in positive person	11.7	64.5	12.0	69.0
Health screening ^c^	0.8	2.5	2.0	1.7
Physical examination of detainee	10.8	5.7	14.9	22.9
Others ^d^	7.6	3.9	13.2	12.5
Detection venues				
Hospital settings	2.4	1.8	5.5	6.6
CDC	4.6	5.3	16.4	22.8
CHCs	1.3	1.2	3.7	2.6
Blood centers	0.7	4.1	2.0	1.3

^a^: Testing ratio, proportion (%) of testing volume to the number of permanent residents. ^b^: Testing of other patients, including testing of inpatient examination, clinical suspects and others in hospitals. ^c^: Health screening, including physical examination of entry-exit personnel, physical examination of personnel in public places, blood donation screening, premarital and recruits physical examination; ^d^: Others, including child testing of female positive persons, occupational exposure testing and paid blood supply testing and others undefined.

**Table 2:** Distribution of baseline CD4 cell count(cells/ul) of HIV newly diagnosed cases in 6 months of diagnosis in Hangzhou, 2007–2018.

Variables	No. (%) of Newly Diagnosed Cases	No. (%) of Subjects with Baseline CD4 Cell Count in 6 Months of Diagnosis	Baseline CD4 Cell Counts (n, %)				Wald c^2^ (*p*)
			<200	200–349	350–499	≥50-	
Total	9400	8341 (88.7)	2389 (28.6)	2433 (29.2)	2058 (24.7)	1461 (17.5)	
Year							
2007–2009	814	549 (67.4)	152 (27.7)	143 (26.0)	137 (25.0)	117 (21.3)	109.937
2010–2012	1836	1536 (83.7)	373 (24.3)	374 (24.3)	444 (28.9)	345 (22.5)	(*p* < 0.001)
2013–2015	3130	2860 (91.4)	823 (28.8)	807 (28.2)	713 (24.9)	517 (18.1)	
2016–2018	3620	3396 (93.8)	1041 (30.7)	1109 (32.7)	764 (22.5)	482 (14.2)	
Age group							
<20	320 (3.4)	278 (86.9)	29 (10.4)	76 (27.3)	104 (37.4)	69 (24.8)	635.425
20~29	3659 (38.9)	3238 (88.5)	567 (17.5)	959 (29.6)	956 (29.5)	756 (23.3)	(*p* < 0.001)
30~39	2441 (26.0)	2143 (87.8)	661 (30.8)	587 (27.4)	505 (23.6)	390 (18.2)	
40~49	1544 (16.4)	1408 (91.2)	562 (39.9)	422 (30.0)	270 (19.2)	154 (10.9)	
≥54	1436 (15.3)	1274 (88.8)	570 (44.7)	389 (30.5)	223 (17.5)	92 (7.2)	
Gender							
Male	8230 (87.6)	7389 (89.8)	2098 (28.4)	2150 (29.1)	1846 (25)	1295 (17.5)	4.028
Female	1170 (12.4)	952 (81.4)	291 (30.6)	283 (29.7)	212 (22.3)	166 (17.4)	(*p* = 0.258)
Marital status							
Single/divorced/widowed	6648 (70.7)	5904 (88.8)	1413 (23.9)	1721 (29.1)	1592 (27)	1178 (20)	268.786
Married	2695 (28.7)	2427 (90.0)	973 (40.1)	709 (29.2)	463 (19.1)	282 (11.6)	(*p* < 0.001)
unknown	57 (0.6)	10 (17.5)	3 (30.0)	3 (30.0)	3 (30.0)	1 (10.0)	
Education							
Primary school or below	1389 (14.7)	1137 (82.9)	446 (39.2)	327 (28.8)	230 (20.2)	134 (11.8)	134.417
Middle or high school	5170 (55.0)	4508 (87.2)	1342 (29.8)	1287 (28.5)	1087 (24.1)	792 (17.6)	(*p* < 0.001)
College or above	2841 (30.2)	2696 (95.0)	601 (22.3)	819 (30.4)	741 (27.5)	535 (19.8)	
Occupation							
Civilian/Enterprise Personnel	702 (7.5)	670 (95.4)	169 (25.2)	197 (29.4)	191 (28.5)	113 (16.9)	249.349
Student	425 (4.5)	408 (96.0)	57 (14.0)	121 (29.7)	132 (32.4)	98 (24)	(*p* < 0.001)
Service workers	3990 (42.4)	3616 (90.6)	961 (26.6)	1038 (28.7)	921 (25.5)	696 (19.2)	
Migrant workers	1673 (17.8)	1475 (88.2)	427 (28.9)	451 (30.6)	362 (24.5)	235 (15.9)	
Farmers	1239 (13.2)	1060 (85.6)	455 (42.9)	313 (29.5)	187 (17.6)	105 (9.9)	
Retired personnel	211 (2.2)	190 (90.0)	86 (45.3)	56 (29.5)	32 (16.8)	16 (8.4)	
Others	1160 (12.3)	922 (79.5)	234 (25.4)	257 (27.9)	233 (25.3)	198 (21.5)	
Ethnicity							
Han	8918 (94.9)	7998 (89.7)	2305 (28.8)	2339 (29.2)	1961 (24.5)	1393 (17.4)	5.509
Minority group	482 (5.1)	343 (71.2)	84 (24.5)	94 (27.4)	97 (28.3)	68 (19.8)	(*p* = 0.138)
Regional categories							
Downtown	8681 (92.4)	7702 (88.8)	2203 (28.6)	2223 (28.9)	1916 (24.9)	1360 (17.7)	6.131
Suburbs	719 (7.6)	639 (88.9)	186 (29.1)	210 (32.9)	142 (22.2)	101 (15.8)	(*p* = 0.105)
Permanent residence							
Hangzhou city	5510	5155 (93.6)	1364 (26.5)	1538 (29.8)	1303 (25.3)	950 (18.4)	32.894
Outside Hangzhou	3890	3186 (81.9)	1025 (32.2)	895 (28.1)	755 (23.7)	511 (16.0)	(*p* < 0.001)
Detection route							
VCT	2044 (21.7)	1901 (93.0)	314 (16.5)	605 (31.8)	569 (29.9)	413 (21.7)	650.907
Pre-test of blood/products receiving	78 (0.8)	59 (75.6)	41 (69.5)	9 (15.3)	7 (11.9)	2 (3.4)	(*p* < 0.001)
STD Clinics	1489 (15.8)	1380 (92.7)	370 (26.8)	414 (30.0)	365 (26.4)	231 (16.7)	
Detection of other patients	1789 (19.0)	1581 (88.4)	649 (41)	421 (26.6)	304 (19.2)	207 (13.1)	
Maternal examination	107 (1.1)	90 (84.1)	13 (14.4)	19 (21.1)	33 (36.7)	25 (27.8)	
Preoperative testing	1982 (21.1)	1728 (87.2)	713 (41.3)	464 (26.9)	328 (19.0)	223 (12.9)	
Spouse or sexual partner testing in positive person	188 (2.0)	168 (89.4)	64 (38.1)	56 (33.3)	26 (15.5)	22 (13.1)	
health screening	492 (5.2)	420 (85.4)	59 (14.0)	113 (26.9)	131 (31.2)	117 (27.9)	
Physical examination of detainee	351 (3.7)	213 (60.7)	31 (14.6)	61 (28.6)	57 (26.8)	64 (30.0)	
others	880 (9.4)	801 (91.0)	135 (16.9)	271 (33.8)	238 (29.7)	157 (19.6)	
Detection venues							
Hospital settings	5751 (61.2)	5059 (88.0)	1833 (36.2)	1380 (27.3)	1111 (22)	735 (14.5)	384.335
CDC	2682 (28.5)	2395 (89.3)	425 (17.7)	768 (32.1)	682 (28.5)	520 (21.7)	(*p* < 0.001)
CHCs	694 (7.4)	653 (94.1)	100 (15.3)	216 (33.1)	192 (29.4)	145 (22.2)	
Blood centers	273 (2.9)	234 (85.7)	31 (13.2)	69 (29.5)	73 (31.2)	61 (26.1)	
Transmission route							
Homosexual contact	5198 (55.3)	4881 (93.9)	1124 (23.0)	1483 (30.4)	1343 (27.5)	931 (19.1)	201.551
Heterosexual contact	3911 (41.6)	3330 (85.1)	1227 (36.8)	915 (27.5)	679 (20.4)	509 (15.3)	(*p* < 0.001)
IDU	160 (1.7)	76 (47.5)	17 (22.4)	20 (26.3)	23 (30.3)	16 (21.1)	
Unknown	131 (1.4)	54 (41.2)	21 (38.9)	15 (27.8)	13 (24.1)	5 (9.3)	

Notes: VCT: HIV Voluntary Counseling & Testing; STD: sexually transmitted diseases; CDC: center for disease control and prevention; CHCs: center for health care services; IDU: people who inject drugs.

**Table 3:** Factors associated with DHD, based on multivariate logistic regression analysis.

Variables	No. of Subjects with Baseline CD4 Cell Count within 6 Months of Diagnosis	No. (%) of Subjects with DHD	cOR (95% CI)	*p*	aOR (95% CI)	*p*
Total	8341	2423 (29.0)				
Year						
2007–2009	549	156 (28.4)	reference		reference	
2010–2012	1536	386 (25.1)	0.85 (0.68, 1.05)	0.132	0.85 (0.67, 1.07)	0.162
2013–2015	2860	833 (29.1)	1.04 (0.85, 1.27)	0.737	1.06 (0.85, 1.32)	0.603
2016–2018	3396	1048 (30.9)	1.12 (0.92, 1.37)	0.249	1.02 (0.82, 1.27)	0.83
Age group						
<20	278	29 (10.4)	reference		reference	
20~29	3238	573 (17.7)	1.85 (1.24–2.74)	0.002	1.85 (1.21–2.83)	0.004
30~39	2143	670 (31.3)	3.91 (2.63–5.79)	<0.001	3.64 (2.36–5.61)	<0.001
40~49	1408	573 (40.7)	5.89 (3.95–8.78)	<0.001	4.69 (3.03–7.23)	<0.001
≥50	1274	578 (45.4)	7.13 (4.78–10.64)	<0.001	4.26 (2.72–6.68)	<0.001
Gender						
Female	952	295 (31.0)	reference		reference	
Male	7389	2128 (28.8)	0.90 (0.78, 1.04)	0.162	1.53 (1.29, 1.82)	<0.001
Marital status						
Single/divorced/widowed	5904	1429 (24.2)	reference		reference	
unknown	10	3 (30.0)	1.34 (0.35, 5.20)	0.67	0.57 (0.14, 2.37)	0.44
Married	2427	991 (40.8)	2.16 (1.96, 2.39)	<0.001	1.23 (1.08, 1.39)	<0.001
Education						
Primary school or below	1137	456 (40.1)	reference		reference	
Middle or high school	4508	1362 (30.2)	0.65 (0.57, 0.74)	<0.001	1.00 (0.86–1.18)	0.914
College or above	2696	605 (22.4)	0.43 (0.37, 0.50)	<0.001	0.99 (0.82–1.21)	0.969
Occupation						
Civilian/Enterprise Personnel	670	172 (25.7)	reference		reference	
Student	408	58 (14.2)	0.48 (0.35, 0.67)	<0.001	1.00 (0.69–1.43)	0.996
Service workers	3616	971 (26.9)	1.06 (0.88, 1.28)	0.525	1.09 (0.88–1.35)	0.406
Migrant workers	1475	437 (29.6)	1.22 (0.99, 1.50)	0.06	0.97 (0.77–1.23)	0.834
Farmers	1060	462 (43.6)	2.24 (1.81, 2.76)	<0.001	1.19 (0.93–1.53)	0.159
Retired personnel	190	86 (45.3)	2.39 (1.71, 3.34)	<0.001	1.05 (0.72–1.53)	0.808
Others	922	237 (25.7)	1.00 (0.80, 1.26)	0.988	0.93 (0.72–1.20)	0.932
Ethnicity						
Han	7998	2338 (29.2)	reference		reference	
Minority group	343	85 (24.8)	0.80 (0.62, 1.02)	0.076	0.98 (0.75–1.29)	0.898
Regional categories						
Downtown	7702	2237 (29.0)	reference		reference	
Suburbs	639	186 (29.1)	1.00 (0.84, 1.20)	0.973	0.87 (0.72–1.07)	0.187
permanent residence						
Hangzhou city	5155	1379 (26.8)	reference		reference	
Outside Hangzhou	1265	1044 (32.8)	1.34 (1.21, 1.47)	<0.001	1.23 (1.11, 1.37)	<0.001
Detection route						
Voluntary counselling and testing	1901	319 (16.8)	reference		reference	
Pre-test of receiving blood/products	59	41 (69.5)	11.30 (6.41, 19.92)	<0.001	5.42 (2.95, 9.97)	<0.001
STD Clinic	1380	373 (27.0)	1.84 (1.56, 2.18)	<0.001	1.19 (0.92, 1.52)	0.17
Testing of other patients	1581	656 (41.5)	3.52 (3.01, 4.11)	<0.001	2.08 (1.64, 2.63)	<0.001
Maternal examination	90	15 (16.7)	0.99 (0.56, 1.75)	0.977	1.05 (0.57, 1.94)	0.881
Preoperative testing	1728	723 (41.8)	3.57 (3.06, 4.16)	<0.001	1.83 (1.44, 2.32)	<0.001
Spouse or sexual partner testing in positive person	168	65 (38.7)	3.13 (2.24, 4.37)	<0.001	1.93 (1.34, 2.77)	<0.001
health screening	420	59 (14.0)	0.81 (0.60, 1.09)	0.17	0.88 (0.56, 1.38)	0.567
Physical examination of detainee	213	32 (15.0)	0.88 (0.59, 1.30)	0.514	0.64 (0.42, 0.99)	0.046
others	801	140 (17.5)	1.05 (0.84, 1.31)	0.659	1.13 (0.84,1.52)	0.41
Detection venues						
Hospital settings	5059	1856 (36.7)	reference		reference	
CDC	2395	433 (18.1)	0.38 (0.34, 0.43)	<0.001	0.68 (0.55, 0.83)	<0.001
Community health centers	653	103 (15.8)	0.32 (0.26, 0.40)	<0.001	0.54 (0.39, 0.75)	<0.001
Blood centers	234	31 (13.2)	0.26 (0.18, 0.39)	<0.001	0.59 (0.34, 1.04)	0.068
Transmission route						
Homosexual contact	4881	1138 (23.3)	reference		reference	
Heterosexual contact	3330	1246 (37.4)	1.97 (1.79, 2.17)	<0.001	1.20 (1.06, 1.36)	0.005
Injecting drug use	76	17 (22.4)	0.95 (0.55, 1.63)	0.846	1.08 (0.59, 1.97)	0.814
Unknown	54	22 (40.7)	2.26 (1.31, 3.91)	0.003	1.32 (0.73, 2.39)	0.365

Notes: STD: sexually transmitted diseases; CDC: center for disease control and prevention.

## Data Availability

All of the principal data are included in the results. Additional materials with further details may be obtained from the corresponding author.
